# Supplementary material for: Mapping lesion, structural disconnection, and functional disconnection to symptoms in semantic aphasia
Source: Brain Struct Funct. 2022 Jul 4;227(9):3043–61. doi: 10.1007/s00429-022-02526-6 (PMC9653334; doi:10.1007/s00429-022-02526-6)
Supplement: Supplementary file 1 — Supplementary file1 (DOCX 13168 KB) [file 429_2022_2526_MOESM1_ESM.docx]

Mapping lesion, structural disconnection, and functional disconnection to symptoms in semantic aphasia – Supplementary Materials

*Brain Structure and Function*

Nicholas E. Souter ^a^, Xiuyi Wang ^a, b^, Hannah Thompson ^c^, Katya Krieger-Redwood ^a^, Ajay D. Halai ^d^, Matthew A. Lambon Ralph ^d^, Michel Thiebaut de Schotten ^e, f^, Elizabeth Jefferies ^a^

^a^ Department of Psychology, University of York, York, UK

^b^ CAS Key Laboratory of Behavioral Science, Institute of Psychology, Chinese Academy of Sciences, Beijing, China

^c^ School of Psychology and Clinical Language Sciences, University of Reading, Reading, UK

^d^ MRC Cognition and Brain Sciences Unit, University of Cambridge, Cambridge, UK

^e^ Brain Connectivity and Behaviour Laboratory, Sorbonne Universities, Paris, France

^f^ Groupe d’Imagerie Neurofonctionnelle, Institut des Maladies Neurodégénératives-UMR 5293, CNRS, CEA, University of Bordeaux, Bordeaux, France

**Corresponding Author**: Nicholas E. Souter, University of York, Department of Psychology, York, YO10 5DD
Email: [nes522@york.ac.uk](mailto:nes522@york.ac.uk)

# Supplementary Materials

## Background Neuropsychology

Patients completed a series of background tests probing language, memory, and executive function. Each individual patients’ performance on these tests can be seen in Supplementary Table 1. Of the 15 patients tested, seven showed evidence of impaired word repetition using a subtest from the Psycholinguistic Assessments of Language Processing in Aphasia battery (PALPA; Kay et al., 1992). Of the 17 patients tested, 15 were impaired for category fluency (eight categories), while 16 were impaired for letter fluency (F, A, S). Sixteen of 22 patients presented with impaired forward digit span, while 14 of 19 presented with impaired backward digit span (Wechsler Memory Scale III; Wechsler, 1997). Eight patients presented with impairments in visuospatial processing, as measured by subtests of the Visual Object and Space Processing Battery (VOSP; Warrington & James, 1991). Patients also completed several tests of executive function, including a subtest of the Test of Everyday Attention (Robertson et al., 1994), Raven’s Coloured Progressive Matrices (Raven, 1962), and the Brixton Spatial Anticipation Test (Burgess & Shallice, 1997). All patients completed at least one of these tests, with fourteen showing some evidence of impairment.

As a measure of core semantic ability, participants completed the Cambridge Semantic Battery (Bozeat et al., 2000). Each individual patient’s performance on these tests can be seen in Supplementary Table 2. Of the 20 tested, 16 patients were impaired on the Picture Naming task [Mean (SD) = 54.6% (37.4)], in which they were required to verbally provide the name for a series of black and white line drawings. Though not a part of the Cambridge Semantic Battery, providing phonemic cues as to the correct target label improved all patients’ performance to ceiling or near-ceiling level [Mean (SD) = 75.3% (41.5)]. Of the 21 tested, 12 patients showed impaired performance on Word-Picture Matching [Mean (SD) = 91.5% (9.4)], in which they were required to match one of ten possible line drawings to a verbally provided probe word. The Camel and Cactus Test (CCT) was used a measure of ability to make thematic associations, requiring matching a probe word/picture to one of four possible targets. 21 patients completed the full version of these tasks, while the remaining two (P22 and P23) completed shortened versions. Of those who completed the full task, 18 patients were impaired on the word version of the CCT [Mean (SD) = 74.1% (18.3)], while 12 were impaired on the picture version [Mean (SD) = 74.5% (21.8)]. The two patients who completed the short versions of the CCT were impaired on both the word and pictures versions.

The ambiguity task (Noonan et al., 2010) required patients to make thematic associations between a probe word and one of three possible targets. Each probe word was a homonym with a dominant (e.g., PEN – PENCIL) and subordinate (e.g., PEN – PIG) association. The latter is believed to tax semantic control more than the former, due to the need to flexibly retrieve non-dominant semantic information (Thompson et al., 2017). Probe words were either presented with no cue, with a contextual cue alluding to the correct target meaning of the word (e.g., PEN – PIG: “the labourers cleaned out the pen”), or with a miscue, alluding to the incorrect interpretation (e.g., PEN – PIG: “he signed his name with a fountain pen”). Twenty-one patients completed the no cue version of the task, with 14 also completing the cue and miscue versions. In the no cue condition, patients performed better for dominant [Mean (SD) = 79.0% (13.8)] than subordinate trials [Mean (SD) = 53.5% (15.1)]. Relative to no cue, cued trials improved performance on subordinate [Mean (SD) = 71.9% (15.6)] but not dominant trials [Mean (SD) = 77.4% (14.6)]. Miscued trials considerably impaired accuracy on dominant [Mean (SD) = 61.2% (21.3)], and somewhat on subordinate trials [Mean (SD) = 45.0% (19.9)]. Contextual cues therefore improved accuracy on the most difficult trials, while contextual miscues impaired performance on the easiest trials.

The synonym judgement task (Samson et al., 2007) required participants to match a probe word to a possible synonym, presented alongside two foils. In each trial, one of these foils acted as either a strong (e.g., probe: DESERT, target: WILDERNESS, distractor: SAND) or weak (e.g., probe: HAZARD, target: DANGER, distractor: LIGHT) thematic distractor. Strong thematic distractors should impair performance to a greater extent than weak distractors, as SA patients are strongly influenced by irrelevant but competing information (Jefferies, 2013). Sixteen patients were tested on this measure. Overall, the sample performed better on weak distractor trials [Mean (SD) = 69.3% (13.4)] than strong distractor trials [Mean (SD) = 49.6% (16.6)]. All but one patient (P13) showed this expected pattern.

The object use task (Corbett et al., 2011) provides a non-verbal measure of semantic control. Herein, patients are required to identify the appropriate object, of six possible options, to perform a given action (e.g., “Crack a nut”). The target objects could be either be ‘canonical’ such that they are typically used to complete this action (e.g., NUT CRACKER), or an ‘alternative’ object which could be used to complete the action if necessary (e.g., HAMMER). Alternative trials should require greater semantic control as they require access to non-dominant information about the target object, and inhibition of dominant information (e.g., that hammers are typically used in construction). Twenty patients were tested on this measure. Overall, the sample performed better on canonical [Mean (SD) = 92.7% (7.5)] than on alternative trials [Mean (SD) = 59.5% (19.7)]. This was true for all 20 patients.

### *Supplementary Table 1. Patient performance on background neuropsychological testing.*

|  | Language | | | Verbal working memory | | Executive | | | | Visual Object and Space Processing battery | | | |
| --- | --- | --- | --- | --- | --- | --- | --- | --- | --- | --- | --- | --- | --- |
|  | PALA 9 Word repetition | Category Fluency | Letter Fluency | Forwards digit span | Backwards digit span | Brixton | Ravens | TEA without distraction | TEA with distraction | Dot counting | Position discrimination | Number location | Cube analysis |
| Max | 80 | - | - | 8 | 7 | 54 | 36 | 7 | 10 | 10 | 20 | 10 | 10 |
| Cut-off | 73 | 62 | 18 | 5.54 | 3.66 | 28 | 28 ^a^ | 4.2 | 2.6 | 8 | 18 | 7 | 6 |
| Mean | 62.2 | 32.3 | 7.8 | 3.6 | 1.8 | 23.7 | 26.0 | 5.0 | 4.1 | 8.7 | 18.4 | 8.3 | 6.8 |
| P01 | **68.8** | **49** | **14** | 6 | **2** | 28 | 20 | 7 | 9 | 8 | 19 | 9 | **4** |
| P02 | **64** | **18** | **0** | **4** | 2 | **7** | **12** | 6 | 3 | 10 | 18 | 9 | **3** |
| P03 | 75.2 | **24** | 19 | 8 | 4 | 28 | 31 | 5 | 9 | NT | NT | NT | NT |
| P04 | 76.8 | **11** | **8** | **4** | **1** | **14** | **6** | **2** | 3 | **6** | **16** | 8 | **4** |
| P05 | 80 | **25** | **14** | 6 | **3** | **11** | **13** | 7 | 9 | **3** | **15** | **2** | **4** |
| P06 | **64.8** | **25** | **5** | **3** | 2 | 34 | 26 | **3** | **2** | 10 | 20 | 10 | **5** |
| P07 | NT | **61** | **13** | **5** | 3 | 37 | 29 | 5 | 6 | NT | NT | NT | NT |
| P08 | NT | NT | NT | **0** | **0** | 34 | **24** | 5 | **1** | 8 | 20 | 8 | 9 |
| P09 | 75 | **26** | **2** | 5 | **2** | **26** | **24** | 5 | **1** | 9 | 19 | 10 | **4** |
| P10 | **42** | **15** | **2** | **1** | **0** | **18** | 31 | 6 | **1** | 10 | **15** | **5** | **4** |
| P11 | **1** | NT | NT | **2** | NT | 31 | 34 | 7 | 7 | 8 | 19 | 10 | 10 |
| P12 | **71** | **26** | **2** | **4** | **2** | **7** | **27** | 5 | 3 | 10 | 20 | 10 | 9 |
| P13 | **7** | 69 | **12** | 6 | 4 | 39 | 33 | 5 | 3 | 10 | 20 | 8 | 8 |
| P14 | 74 | 80 | **16** | **4** | **2** | 31 | **21** | 5 | **2** | 10 | 20 | **5** | 10 |
| P15 | NT | NT | NT | **0** | **0** | **21** | 31 | **2** | **1** | **7** | 19 | 8 | 8 |
| P16 | NT | **4** | **3** | **3** | NT | NT | 31 | 7 | 3 | NT | NT | NT | NT |
| P17 | 79 | **26** | **6** | **4** | **0** | **23** | 30 | NT | NT | 10 | 20 | 10 | 9 |
| P18 | 77 | **57** | **9** | **4** | **3** | 30 | 33 | 7 | 6 | 10 | 20 | 10 | 10 |
| P19 | NT | NT | NT | NT | NT | **6** | 32 | NT | NT | NT | **16** | 9 | NT |
| P20 | 78 | **14** | **3** | 6 | **2** | **24** | **19** | **4** | **2** | 10 | **17** | 10 | 7 |
| P21 | NT | **19** | **5** | **3** | **3** | 30 | **25** | **2** | 6 | 9 | 18 | 9 | 8 |
| P22 | NT | NT | NT | **0** | **0** | 16 | 35 | NT | NT | NT | NT | NT | NT |
| P23 | NT | NT | NT | **2** | NT | 27 | 30 | NT | NT | NT | NT | NT | NT |
| # Tested | 15 | 17 | 17 | 22 | 19 | 22 | 23 | 19 | 19 | 17 | 18 | 18 | 17 |
| # Impaired | 7 | 15 | 16 | 16 | 14 | 8 | 9 | 5 | 7 | 3 | 5 | 3 | 7 |

*Note.* Scores are number of correct responses unless otherwise specified. NT = unavailable for testing; TEA = Test of Everyday Attention, elevator counting subtest; VOSP = Visual Object and Space Processing battery. Category fluency corresponds to 8 categories (animals, fruit, birds, breeds of dog, household objects, tools, vehicles, types of boat). Letter fluency corresponds to F, A, S. Cut-offs for impairment correspond to two standard deviations below control mean performance, with impaired scores underlined and in bold. These are taken from control norms from respective tests manuals, unless otherwise specified (see below).

^a^ Cut-offs taken from control testing at the University of York. Number of controls = 20.

### *Supplementary Table 2. Patient performance on the Cambridge Semantic Battery and tests of semantic control.*

|  | Semantic cognition composite score | Picture Naming | | Word-picture matching | CCT | | Ambiguity | | | | | | Synonym with distractors | | Object use | |
| --- | --- | --- | --- | --- | --- | --- | --- | --- | --- | --- | --- | --- | --- | --- | --- | --- |
|  |  | No cues | With cues |  | Word | Picture | Miscued dominant | Miscued subordinate | No cue dominant | No cue subordinate | Cued dominant | Cued subordinate | Strong distractor | Weak distractor | Alternative | Canonical |
| Max | - | 64 | 64 | 64 | 64 | 64 | 30 | 30 | 30 | 30 | 30 | 30 | 42 | 42 | 37 | 37 |
| Cut-off | - | 59 | - | 62.7 | 56.6 | 52.7 | 30 | 26.6 | 28.4 | 27.6 | 30 | 28.8 | 35.4 | 40.4 | 33.7 | 35.9 |
| Mean | - | 35.0 | 48.2 | 58.6 | 47.4 | 47.7 | 18.4 | 13.5 | 23.7 | 16.1 | 23.2 | 21.6 | 20.8 | 29.1 | 22.0 | 34.3 |
| P01 | .76 | **51** | NT | **50** | **54** | 54 | NT | NT | **26** | **23** | NT | NT | NT | NT | **24** | 35 |
| P02 | -.71 | **30** | NT | **54** | **41** | **46** | NT | NT | **19** | **10** | NT | NT | NT | NT | NT | NT |
| P03 | -.51 | **21** | NT | **46** | **42** | **44** | NT | NT | **21** | **13** | NT | NT | NT | NT | **12** | **30** |
| P04 | -2.62 | **5** | NT | **48** | **16** | **15** | **5** | **7** | **11** | **10** | **12** | **14** | **15** | **18** | **9** | **31** |
| P05 | -1.62 | **5** | NT | **50** | **33** | **13** | **19** | **9** | **23** | **10** | **24** | **17** | **18** | **34** | **13** | **31** |
| P06 | -.72 | **55** | NT | **60** | **39** | **36** | **18** | **10** | **23** | **13** | **22** | **22** | **19** | **24** | **24** | 37 |
| P07 | 1.30 | 62 | NT | 64 | 60 | 61 | NT | NT | 29 | **24** | NT | NT | **29** | **36** | **31** | 37 |
| P08 | .66 | **0** | 0 | **56** | **56** | 61 | NT | NT | **25** | **16** | NT | NT | **16** | **33** | **22** | 35 |
| P09 | .22 | **50** | 63 | 64 | **53** | 56 | **14** | **8** | **22** | **14** | **22** | **18** | **20** | **24** | **21** | 35 |
| P10 | -.72 | **19** | 58 | **60** | **29** | **45** | **13** | **14** | **24** | **14** | **19** | **20** | **13** | **29** | **14** | **29** |
| P11 | .73 | **3** | 10 | **52** | 57 | 54 | **21** | **18** | **27** | **19** | **23** | **24** | **30** | **31** | **22** | **33** |
| P12 | -.78 | 61 | 63 | **62** | **43** | **44** | **13** | **10** | **18** | **9** | **21** | **14** | **12** | **23** | **13** | **31** |
| P13 | .96 | **46** | 64 | 63 | **56** | 61 | **26** | 28 | **27** | **21** | **29** | **28** | 38 | **36** | **32** | 37 |
| P14 | .96 | **56** | 62 | 64 | 61 | 53 | **24** | **18** | **28** | **21** | **27** | **23** | **22** | **28** | **26** | 37 |
| P15 | -.98 | **1** | 3 | 63 | **39** | **31** | **12** | **7** | **22** | **11** | **23** | **25** | **15** | **25** | **14** | **32** |
| P16 | .24 | **50** | 63 | 63 | **48** | **51** | **27** | **16** | **25** | **18** | **27** | **25** | NT | NT | **27** | 37 |
| P17 | .52 | **50** | 64 | **62** | **52** | 57 | **19** | **15** | **26** | **17** | **23** | **28** | **23** | **30** | **22** | 35 |
| P18 | 1.04 | 62 | 64 | **62** | 60 | 61 | **26** | **19** | **28** | **19** | **29** | **25** | **17** | **39** | **29** | 37 |
| P19 | .55 | NT | NT | 61 | **50** | 59 | NT | NT | **27** | **17** | NT | NT | NT | NT | **24** | **33** |
| P20 | .43 | 60 | 64 | **62** | 59 | **45** | **20** | **10** | **24** | **19** | **24** | **19** | **21** | **27** | 34 | 37 |
| P21 | .28 | **12** | NT | 64 | **48** | 54 | NT | NT | **23** | **19** | **NT** | **NT** | **25** | **29** | **27** | 37 |
| P22 | - | NT | NT | NT | **7** ^a^ | **10** ^a^ | NT | NT | NT | NT | NT | NT | NT | NT | NT | NT |
| P23 | - | NT | NT | NT | **13** ^a^ | **10** ^a^ | NT | NT | NT | NT | NT | NT | NT | NT | NT | NT |
| # Tested | - | 20 | 12 | 21 | 23 | 23 | 14 | 14 | 21 | 21 | 14 | 14 | 16 | 16 | 20 | 20 |
| # Impaired | - | 16 | - | 12 | 18 | 12 | 14 | 13 | 20 | 21 | 14 | 14 | 15 | 16 | 19 | 8 |

*Note.* Scores are number of correct. NT = unavailable for testing, CCT = Camel and Cactus Test. Cut-offs for impairment are taken from testing at the University of York and correspond to two standard deviations below mean control performance, with impaired scores underlined and in bold. Number of controls: CCT, Picture naming, and Word-picture matching = 10, Ambiguity task, Synonym with distractors, Object use = 8. Semantic composite score reflects regression scores derived from principal components analysis, including performance on CTT words, CCT pictures, and the Ambiguity task (no cue: dominant + subordinate). Lower composite scores reflect greater impairment.

^a^ Patients P22 and P23 completed short versions of the CCT tasks, each comprising 25 trials. Cut-off for impairment for the word and picture versions of the task is 20.7 and 19.6, respectively. As these patients do not have scores for the long version of the CCT tasks or the Ambiguity task, they do not have semantic composite scores.

|  | Semantic Cognition Composite Score | Brixton Spatial Anticipation Test |
| --- | --- | --- |
| 1. – 40% Threshold | 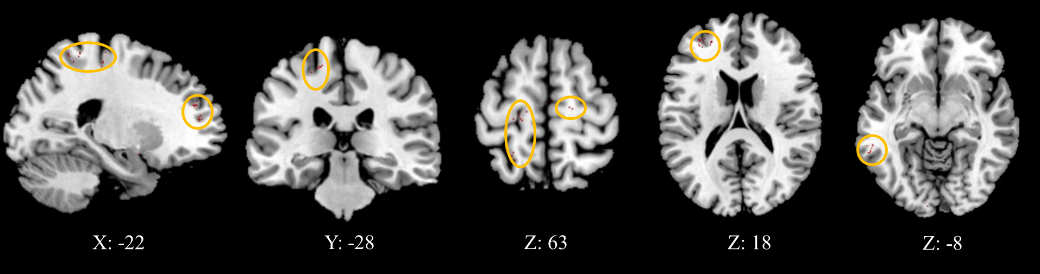 | 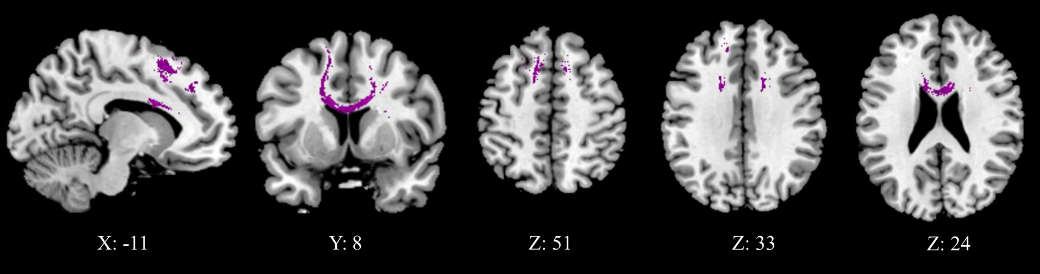 |
| 1. – 50% Threshold | 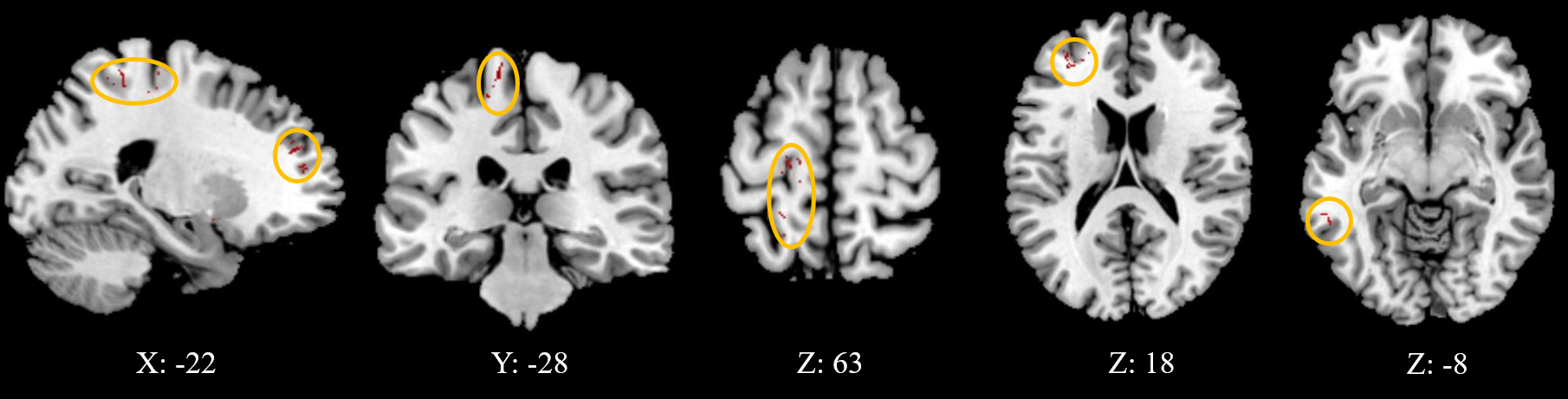 | 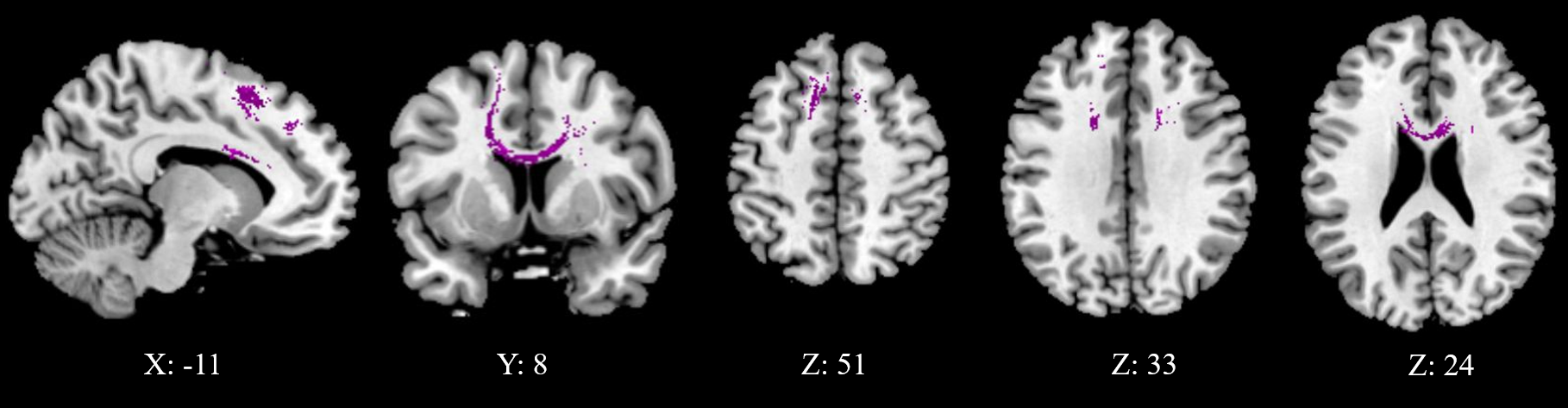 |
| 1. – 60% Threshold | 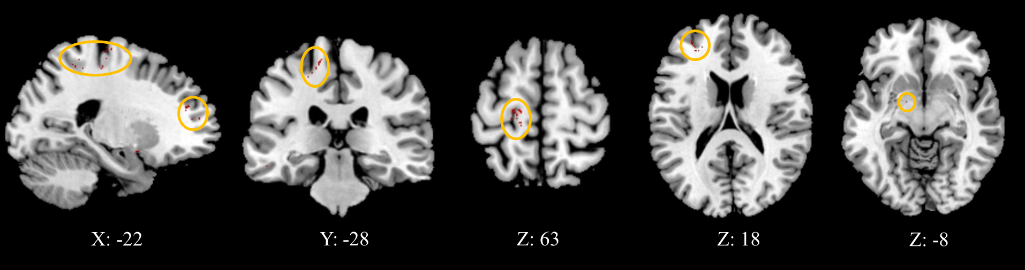 | 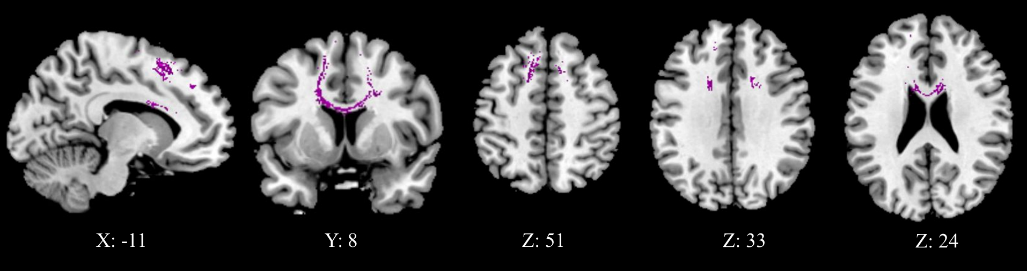 |

## *Supplementary Figure 1. Replication of the structural disconnection symptom mapping from the paper, but at alternative probability thresholds. This includes 40% in Supplementary Figure 1a, 50% in Supplementary Figure 1b, which is the default threshold used in the paper, and 60% in Supplementary Figure 1c. In each case, symptom mapping is presented for patients’ Semantic Cognition Composite Score on the left, and performance on the Brixton Spatial Anticipation Test on the right. Generated using non-parametric permutation tests in Randomise with threshold-free cluster enhancement. Highlighted voxels have a t-value of 2.6 or higher. Small clusters are highlighted in orange circles. 3D rendering generated in SurfIce. Results are consistent at each threshold. For the Semantic Cognition Composite Score, this includes very small voxels located in the left the frontal pole, precentral and postcentral gyri, pMTG, and occipital pole. The only conceptual deviation from the default threshold is a single voxel highlighted in the right precentral gyrus at a threshold of 40%. Clusters are too small to provide clinical significance. For the Brixton Spatial Anticipation Test, at each threshold performance is predicted by structural disconnection across the corpus callosum. N = 20*

| 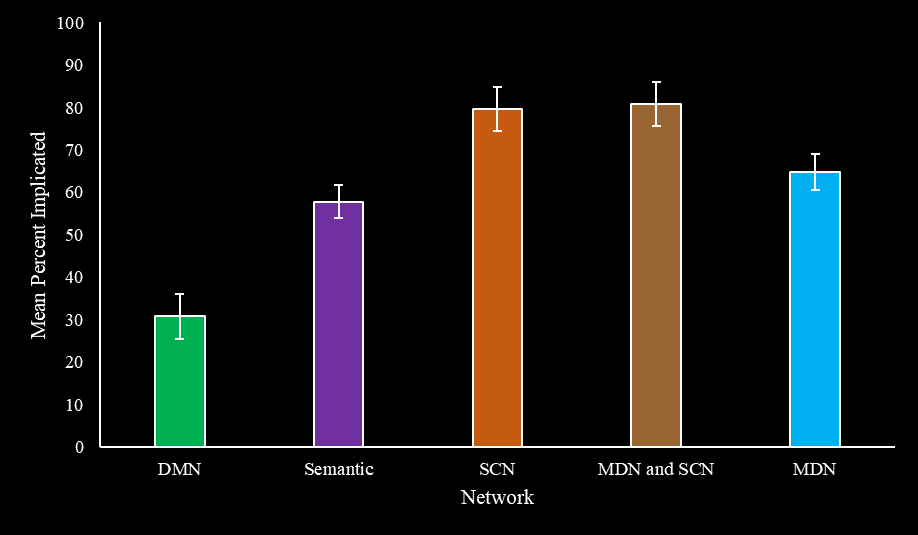  (a) – Mean Percent Functionally Disconnected | |
| --- | --- |
| 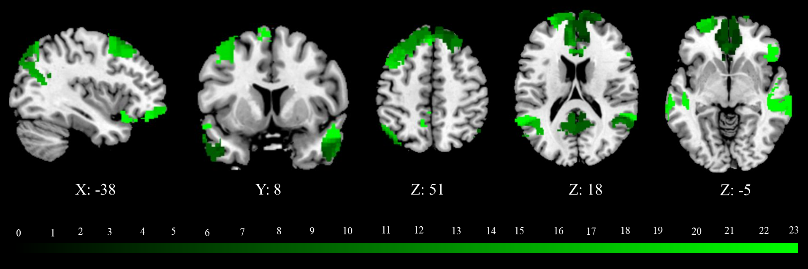  (b) – Default Mode Network | 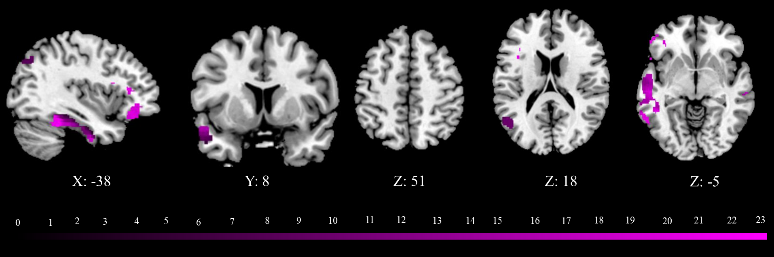  (c) – Semantic (Neurosynth) |
| 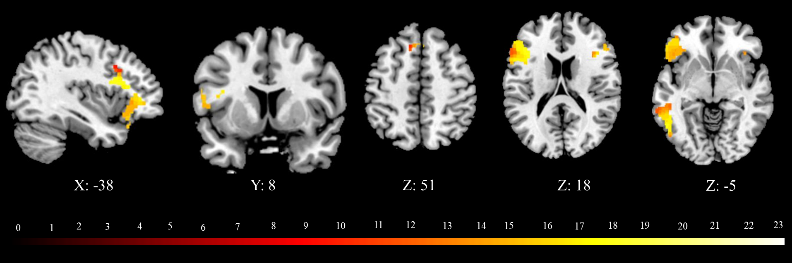  (d) – Semantic Control Network | 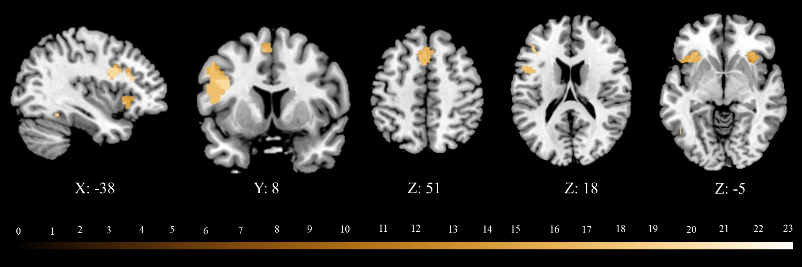  (e) – Multiple Demand Network + Semantic Control Network |
| 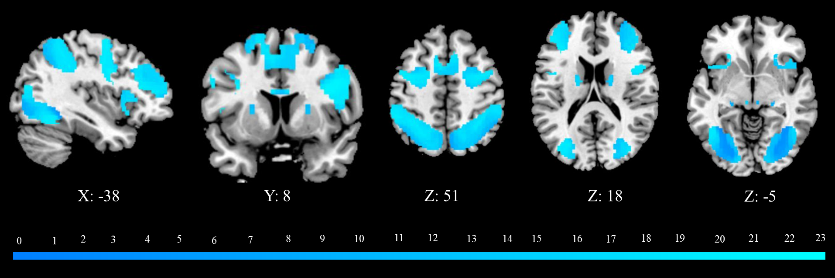  (f) – Multiple Demand Network | |
|  | |

## *Supplementary Figure 2. (a) The mean percent of each network of interest overlapping with patient functional disconnection maps, generated in CONN. DMN = default mode network, SCN = semantic control network, MDN = multiple demand network. This peaks in the semantic control network at 85%, followed by areas shared between the multiple demand and semantic control networks at 81%, core semantic regions at 80%, regions exclusive to the multiple demand network both at 75%, and the default mode network at 44%. Locations of most frequent damage are displayed for each network in following sections. (b) The default mode network, functional disconnection peaks in the right inferior frontal gyrus (pars triangularis), left frontal orbital cortex, left temporooccipital part of the middle temporal gyrus, and left posterior supramarginal gyrus. (c) Core semantic regions, functional disconnection peaks in the left temporooccipital part of the inferior temporal gyrus, left temporooccipital part of the middle temporal gyrus, and left inferior lateral occipital cortex. (d) The semantic control network, functional disconnection peaks in the bilateral inferior frontal gyrus (pars opercularis), and left temporooccipital part of the inferior temporal gyrus. (e) Regions shared by the semantic control and multiple demand networks, functional disconnection peaks in the left precentral gyrus, left inferior frontal gyrus (pars triangularis), and left temporooccipital part of the inferior temporal gyrus. (f) The multiple demand network, functional disconnection peaks in the bilateral precentral gyrus, right inferior frontal gyrus (pars opercularis) and left temporooccipital part of the inferior temporal gyrus. Keys under each map reflects the number of patients whose map overlap in a given voxel. N = 23*

|  | Semantic Cognition Composite | | Brixton Spatial Anticipation Test | |
| --- | --- | --- | --- | --- |
| 1. – Lesion | 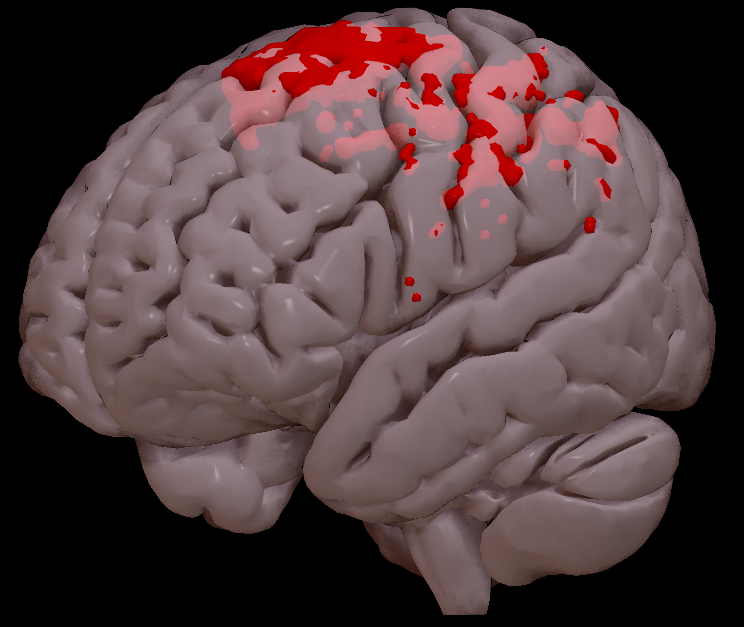 | 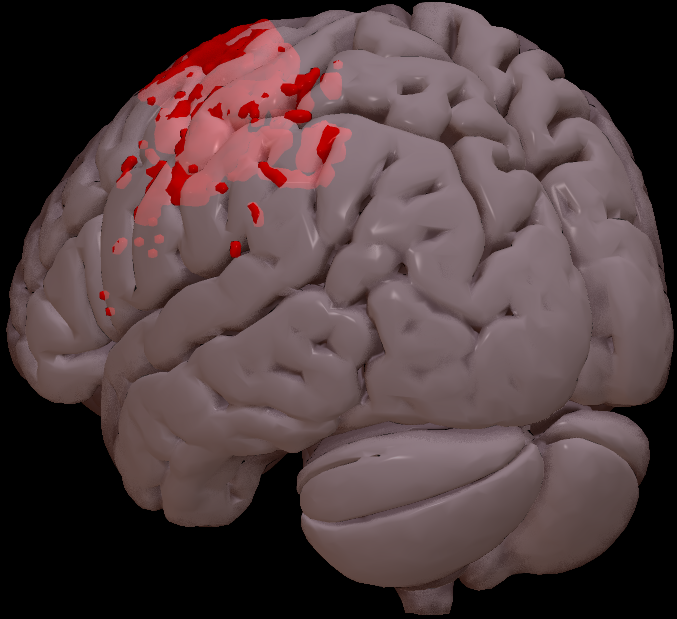 | 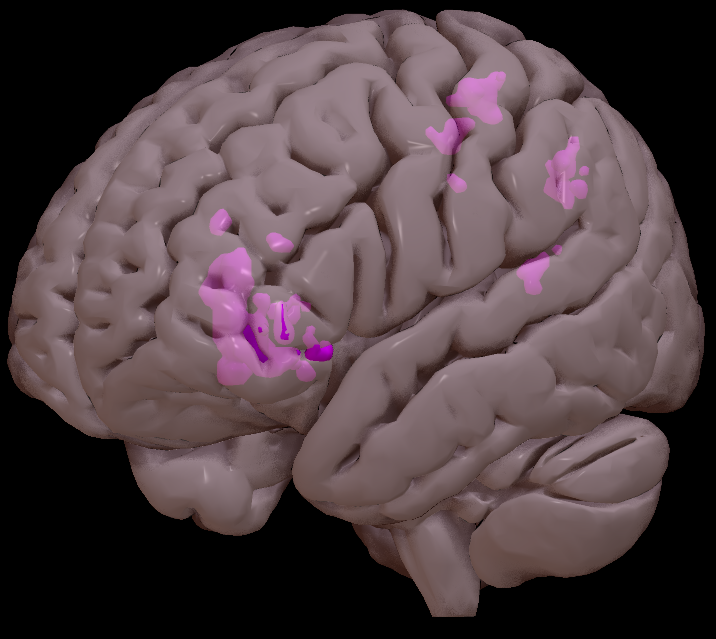 | 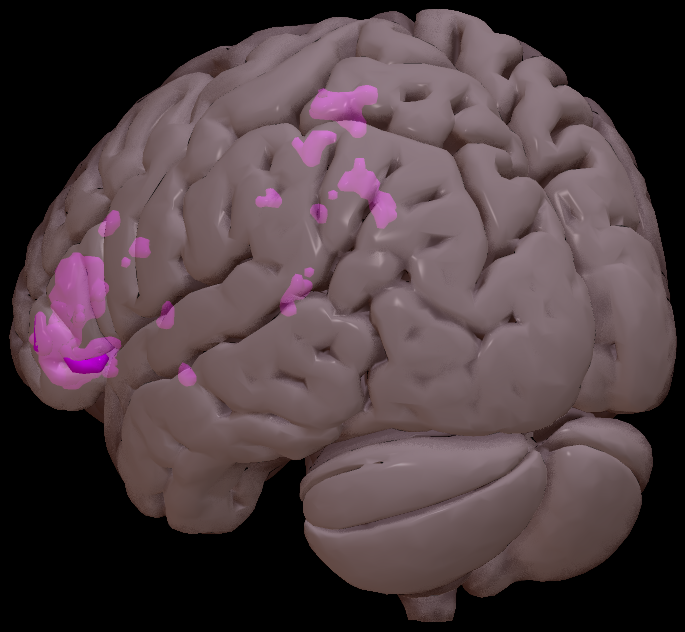 |
|  | 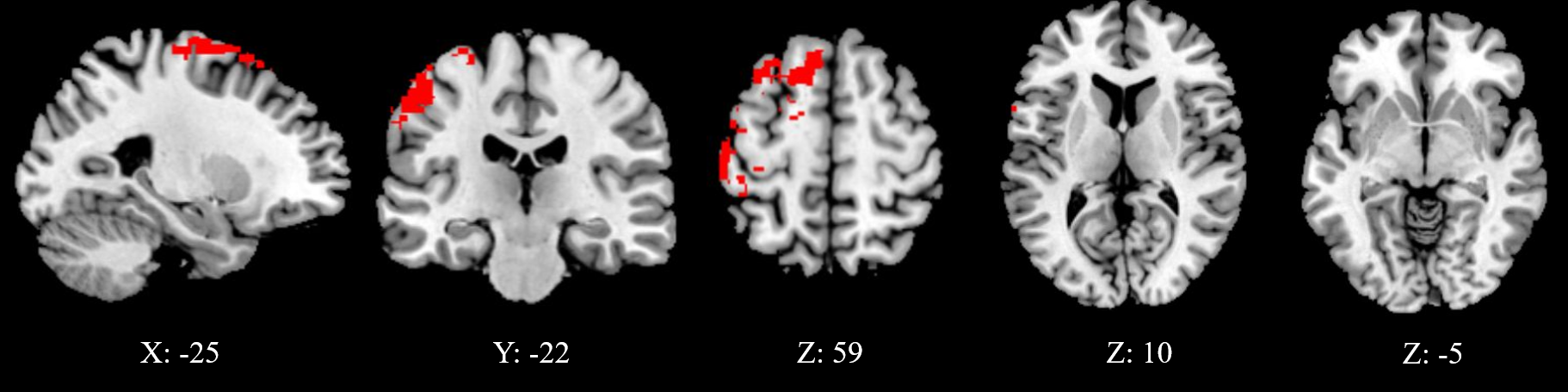 | | 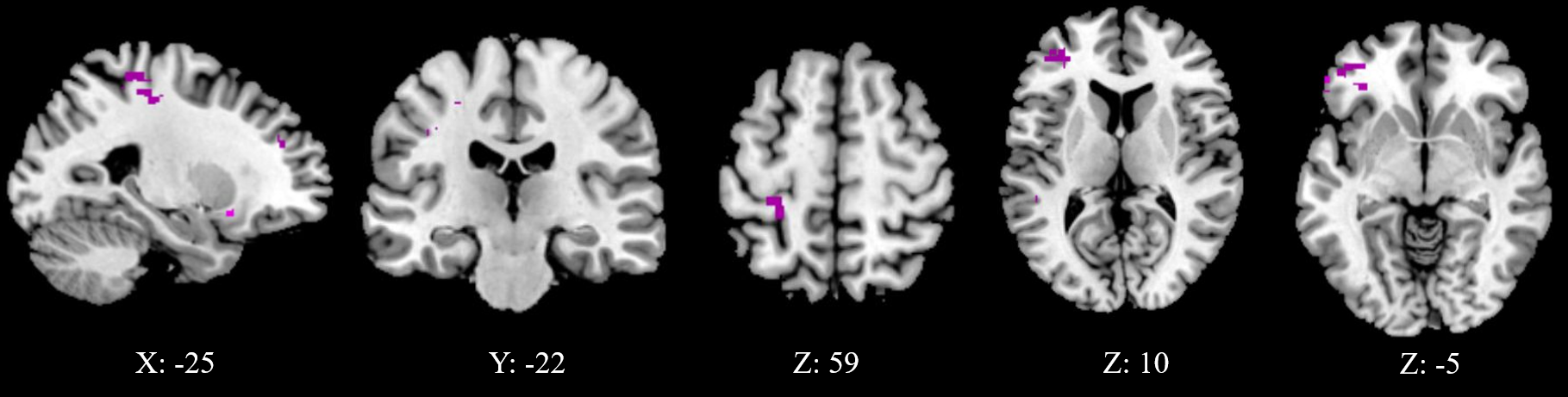 | |
| 1. – Structural Disconnection | 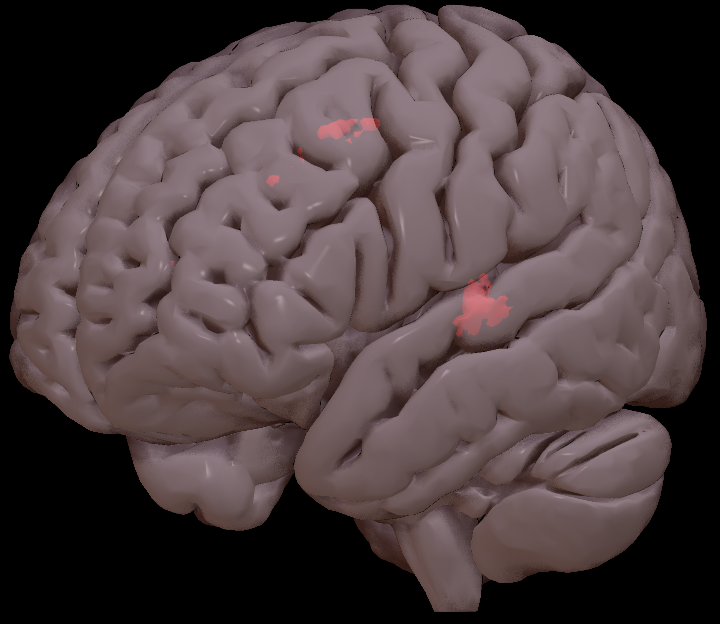 | 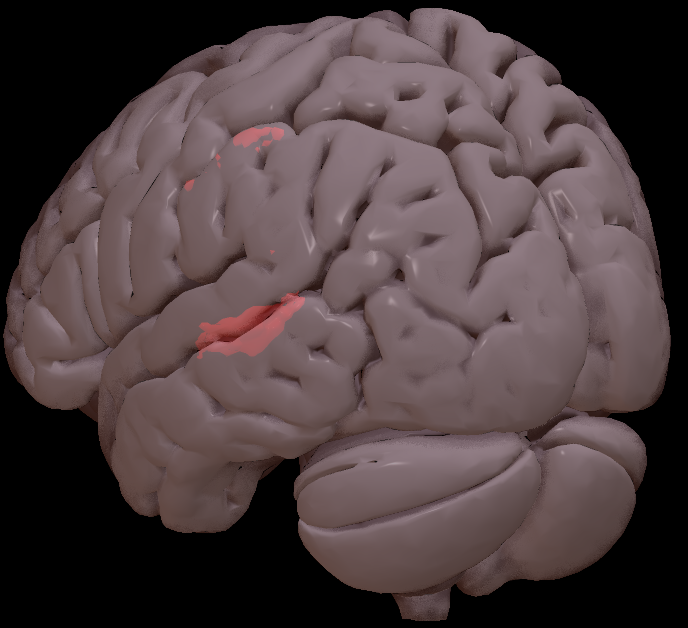 | 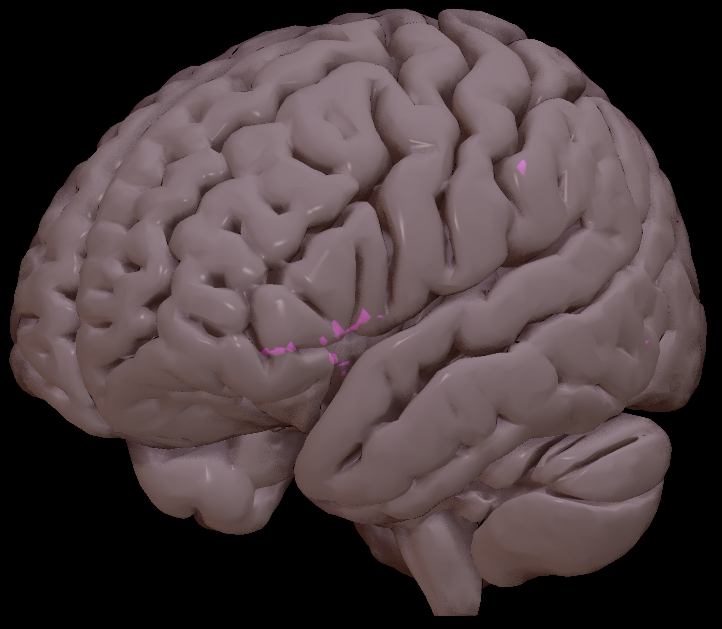 | 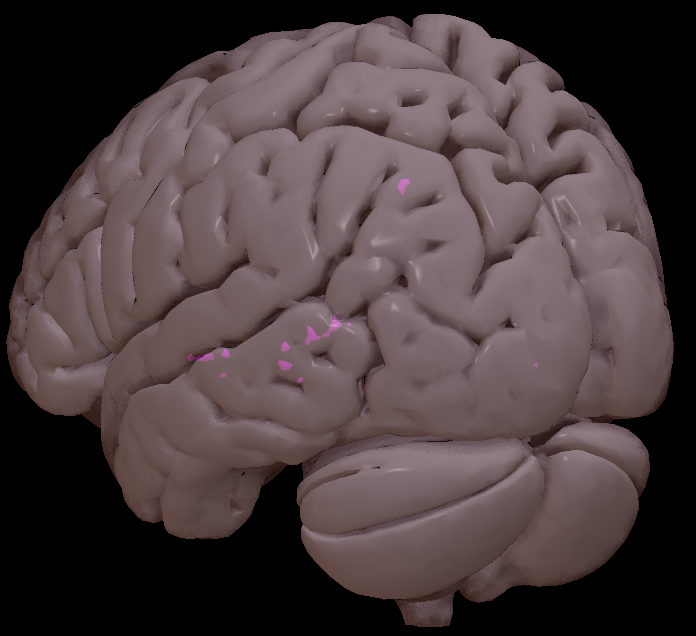 |
|  | 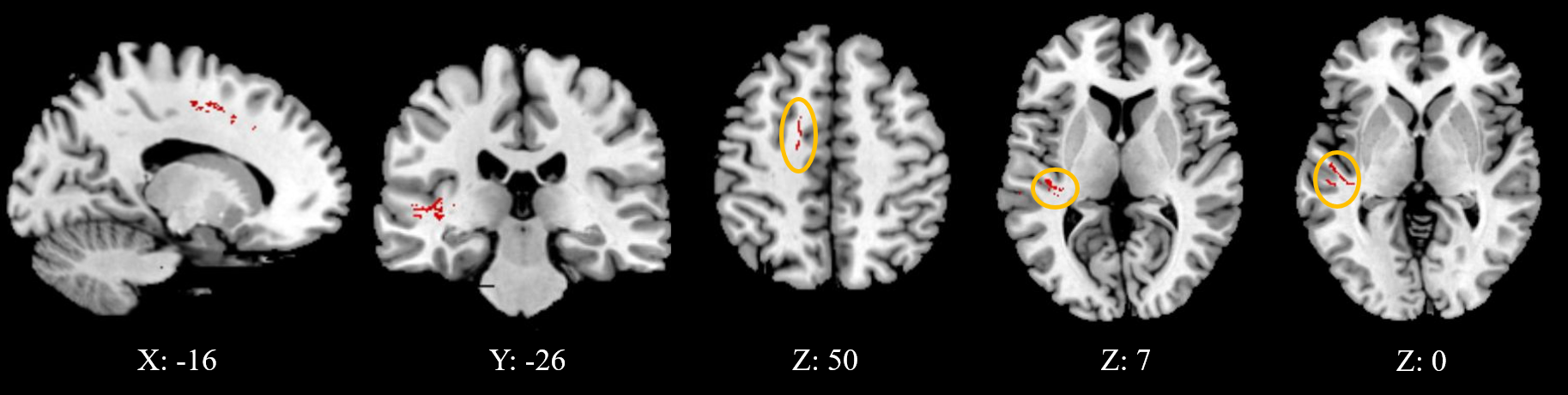 | | 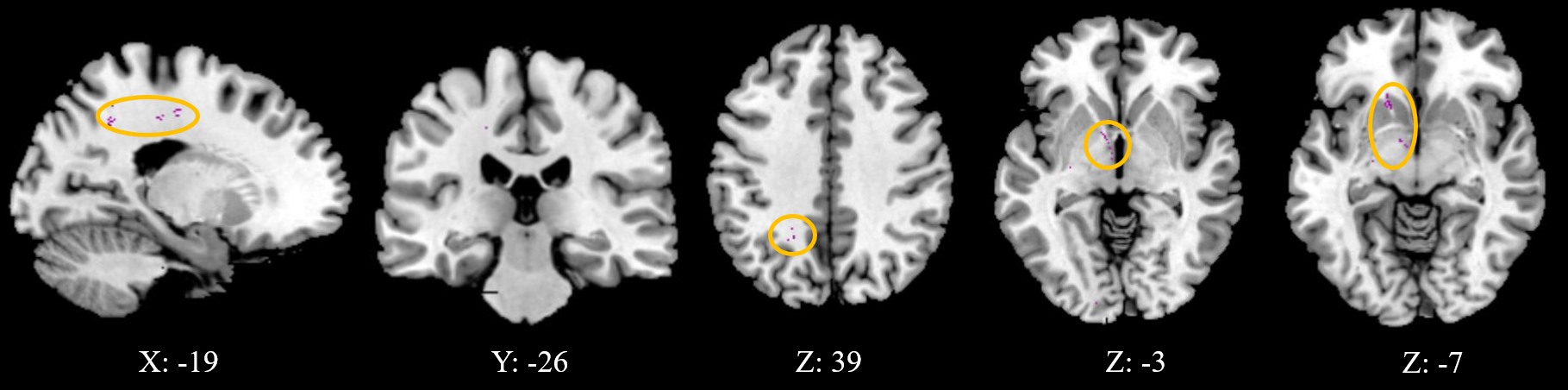 | |
| 1. – Functional Disconnection | 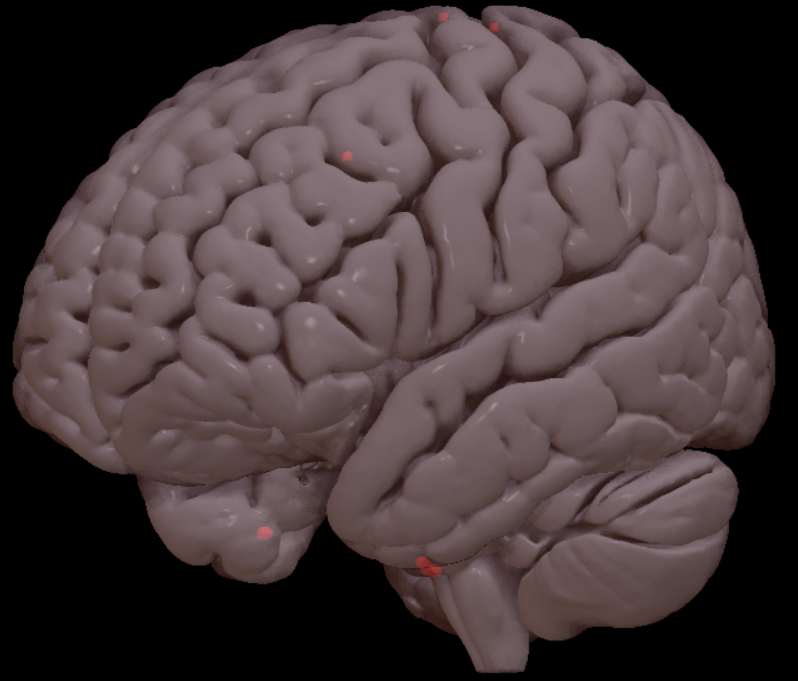 | 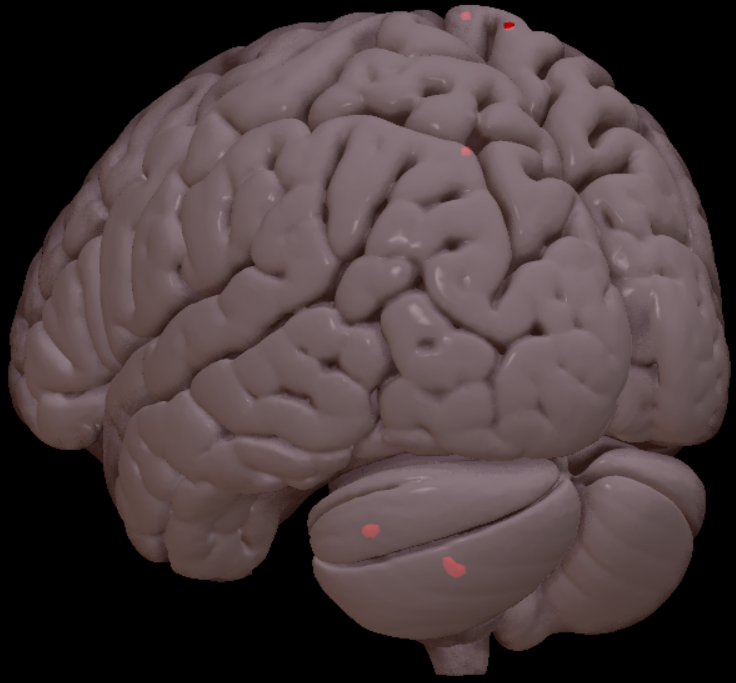 | No voxels met threshold (t > 2.6) | |
|  | 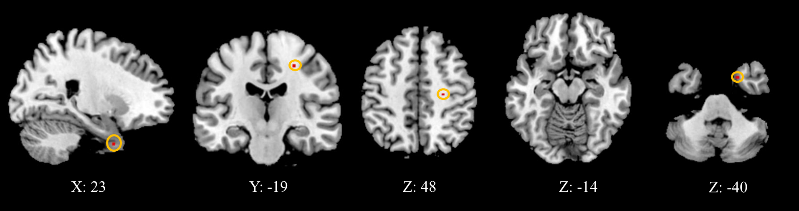 | |  |  |

## *Supplementary Figure 3. Voxels associated with higher semantic cognition composite scores (left) and better performance on the Brixton Spatial Anticipation Test (right), for (a) lesion, (b) structural disconnection, and (c) functional disconnection data. Generated using a non-parametric permutation tests in Randomise with threshold-free cluster enhancement. Highlighted voxels reflect those with a t-value of 2.6 or higher. Small clusters are highlighted in orange circles for visibility. 3D rendering generated in SurfIce. Lesioned clusters associated with better semantic cognition are left fronto-parietal, and implicate the precentral, postcentral, and superior frontal gyri. Lesioned clusters associated with better Brixton performance include the frontal pole, planum temporale, angular gyrus, and postcentral gyrus. Structurally disconnected clusters associated with better semantic cognition include a small bilateral group of voxels in the parietal cortex which do not implicate specific regions or tracts, as well as a cluster in the left Heschl’s gyrus. Structurally disconnected clusters associated with better Brixton performance are similarly sparse, but implicate the putamen, occipital pole, superior parietal lobule and precentral gyrus. Functionally disconnected clusters associated with better semantic cognition include several sparse voxels, including in the brain stem, right temporal pole, and in white matter proximal to the right precentral gyrus. No clusters met the threshold of t > 2.6 for functionally disconnected clusters associated with better Brixton performance. N = 20*

| 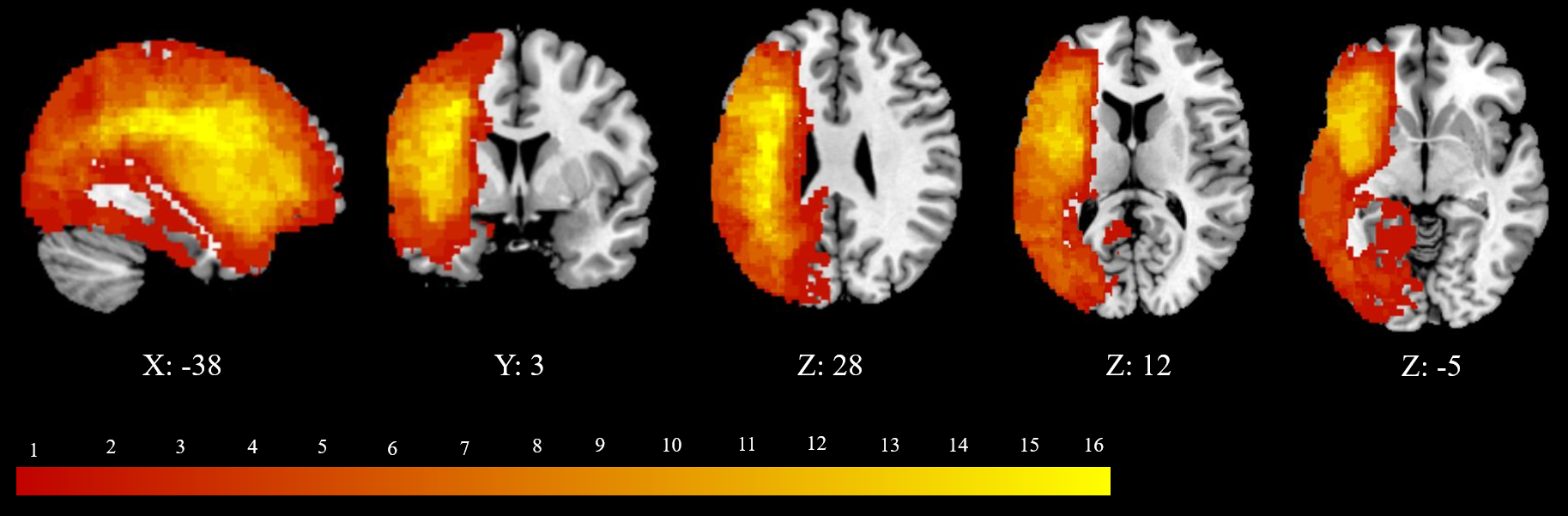 |
| --- |
| – Lesion Overlap Map |
| 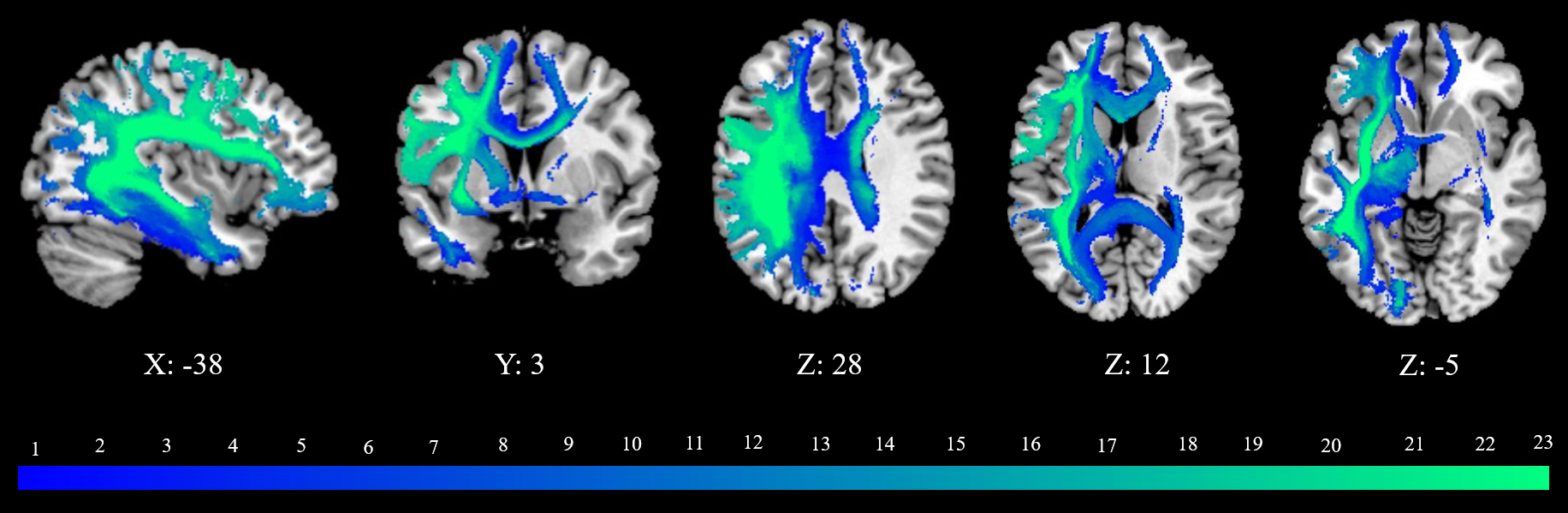 |
| – Structural Disconnection Overlap Map |
| 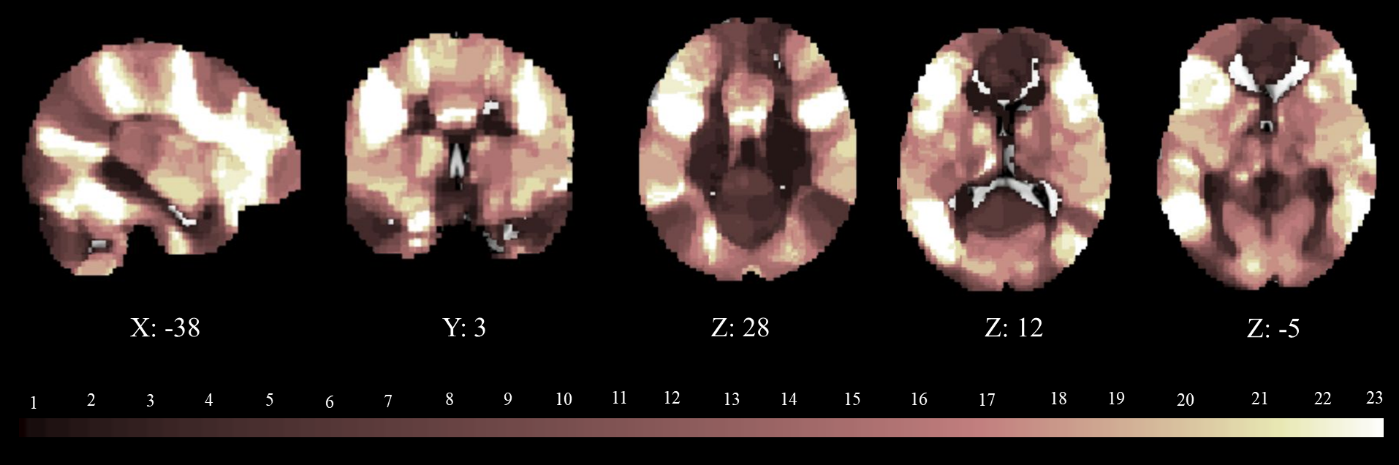 |
| – Functional Disconnection Overlap Map |

## *Supplementary Figure 4. Unthresholded overlap maps for (a) lesion sites, (b) structural disconnection maps, generated using the BCB Toolkit, and (c) functional disconnection maps, generated using CONN. Lesions are confined to the left hemisphere and subsume much of the cortex, affecting each lobe, peaking in the precentral and middle frontal gyri. Maximum overlap is 16 cases. Structural disconnection is largely left lateralised but with some spreading to the right hemisphere. Most left hemisphere white matter is implicated here, but this peaks in the left superior longitudinal fasciculus and inferior fronto-occipital fasciculus. Maximum overlap is all 23 cases. Functional disconnection is bilateral and extensive, subsuming almost the entirety of the brain. This disconnection peaks in the left temporooccipital part of the inferior temporal gyrus, and right inferior frontal gyrus (pars opercularis). Maximum overlap is all 23 cases. N = 23*

| 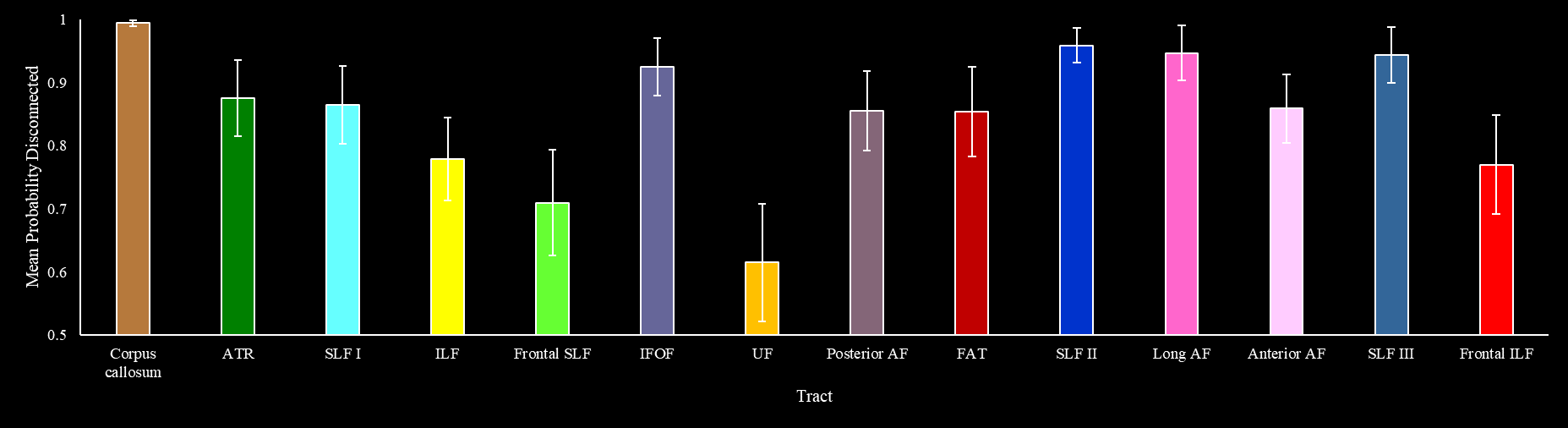 (a) – Mean Probability of Disconnection | |
| --- | --- |
| 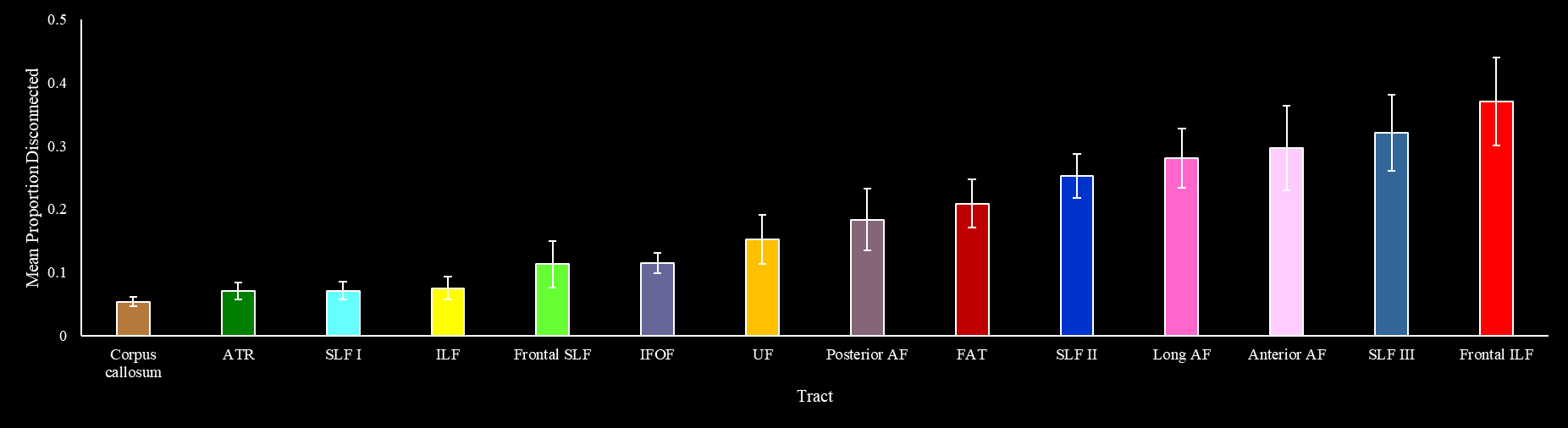 (b) – Mean Proportion Disconnected | |
| 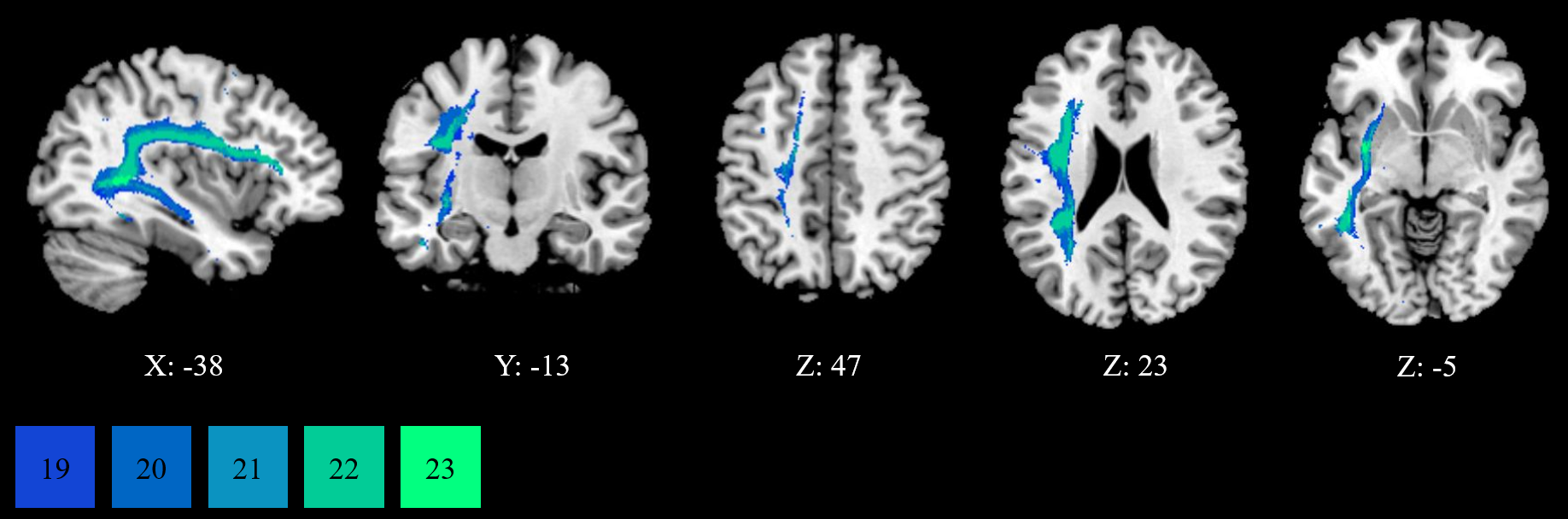 (c) – Sructural Disconnection Overlap Map | |
| 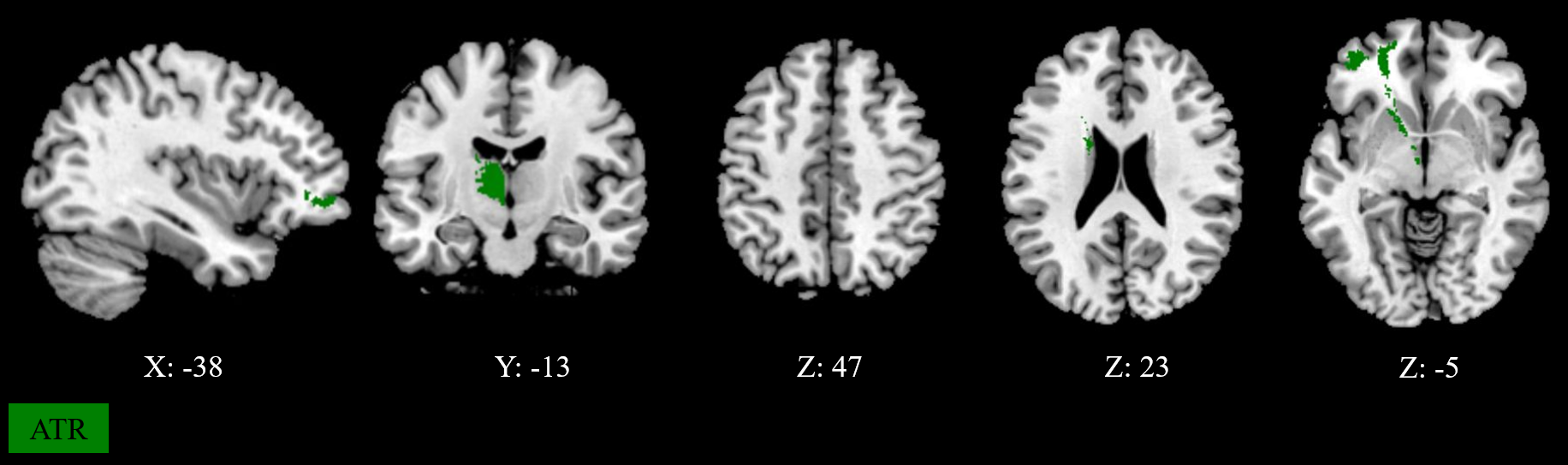 (d) – Anterior Thalamic Radiation | 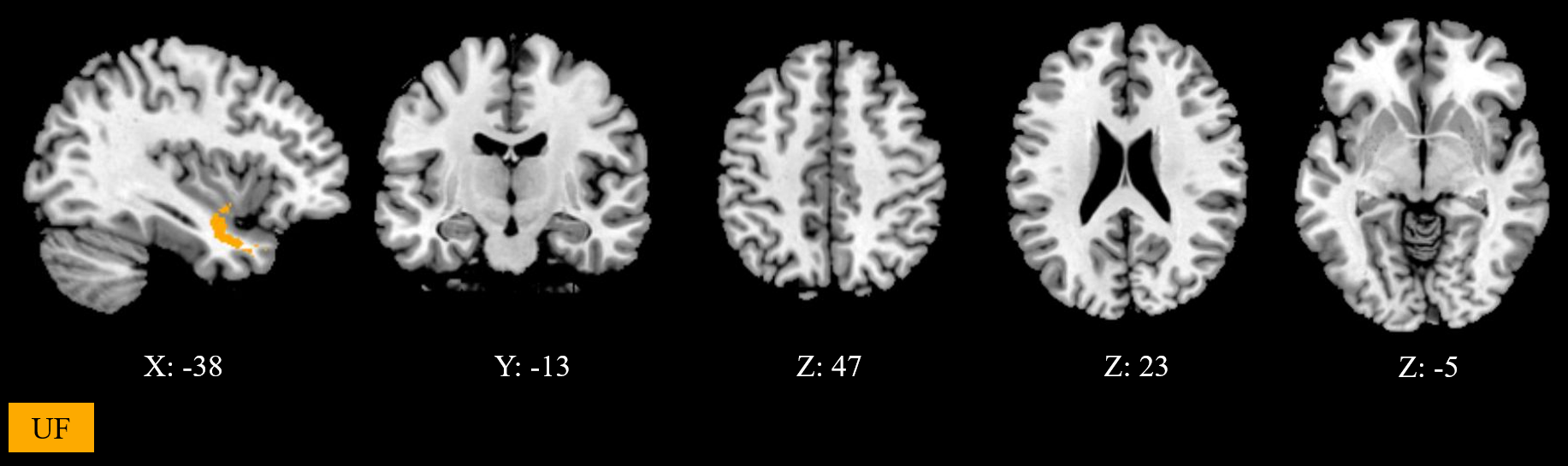 (e) – Uncinate Fasciculus |
| 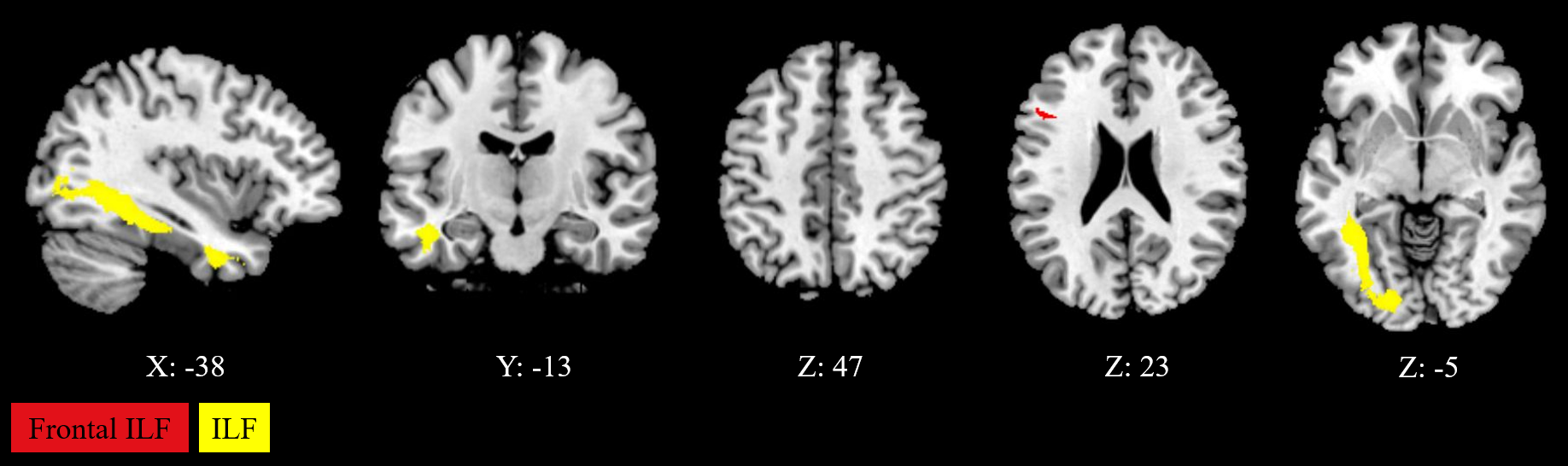 (f) – Inferior Longitudinal Fasciculus | 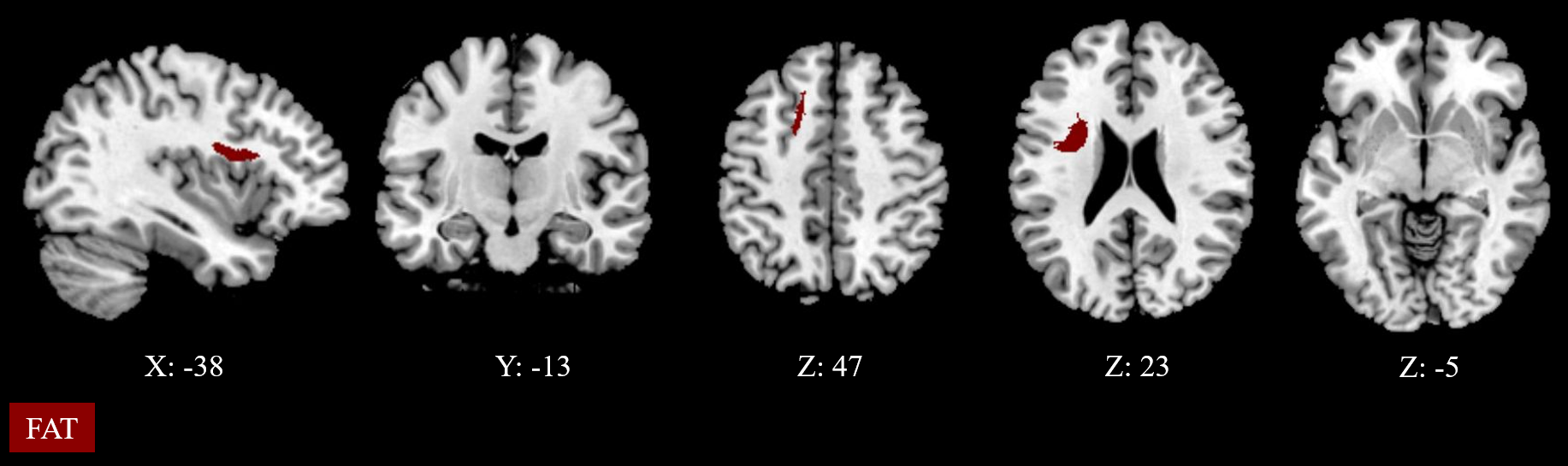 (g) – Frontal Aslant Tract |
| 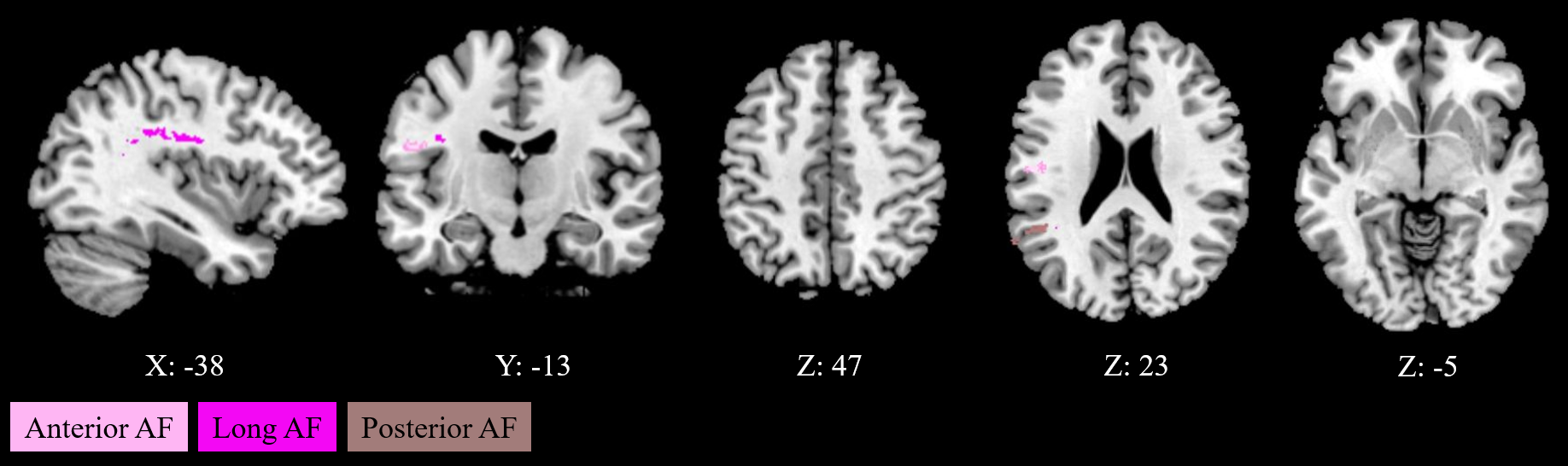 (h) – Arcuate Fasciculus | 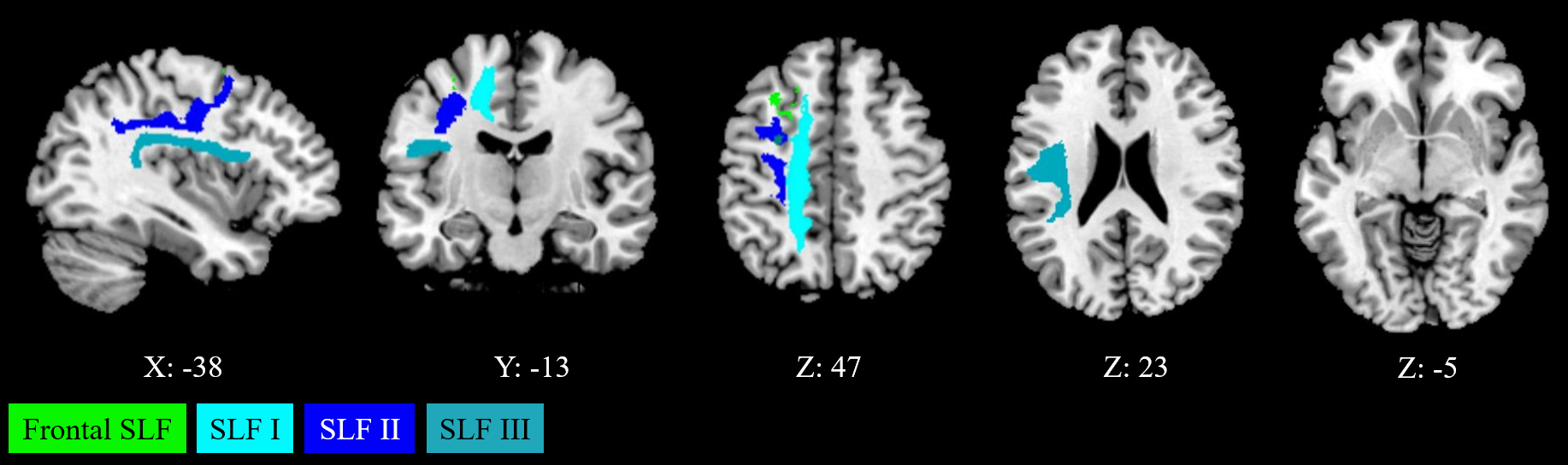 (i) – Superior Longitudinal Fasciculus |
| 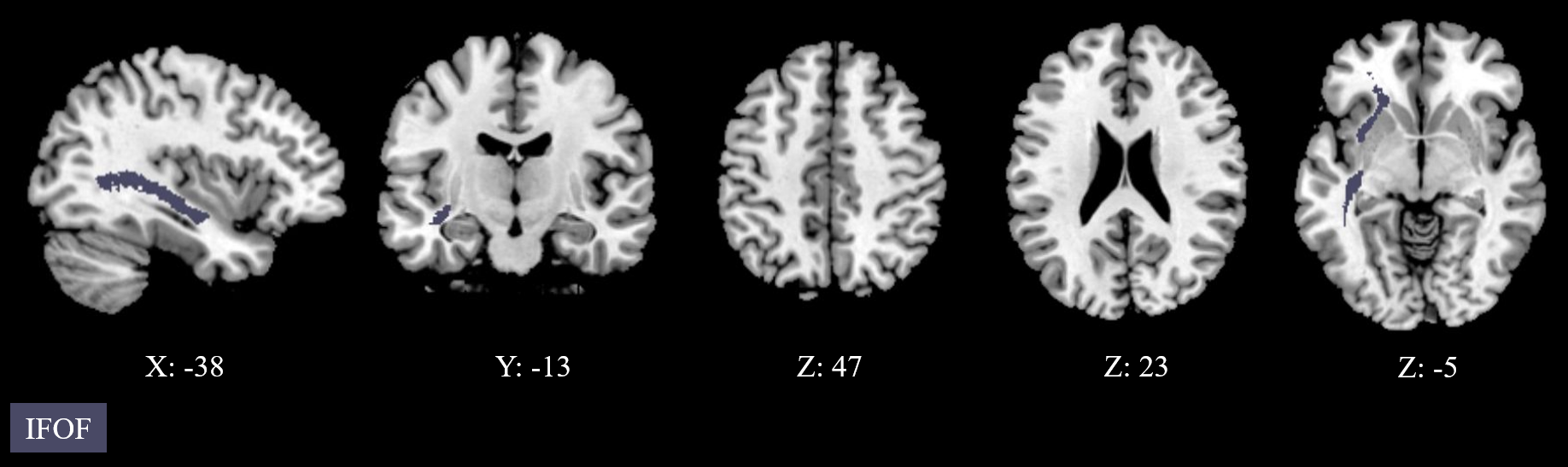 (j) – Inferior Fronto-Occipital Fasciculus | 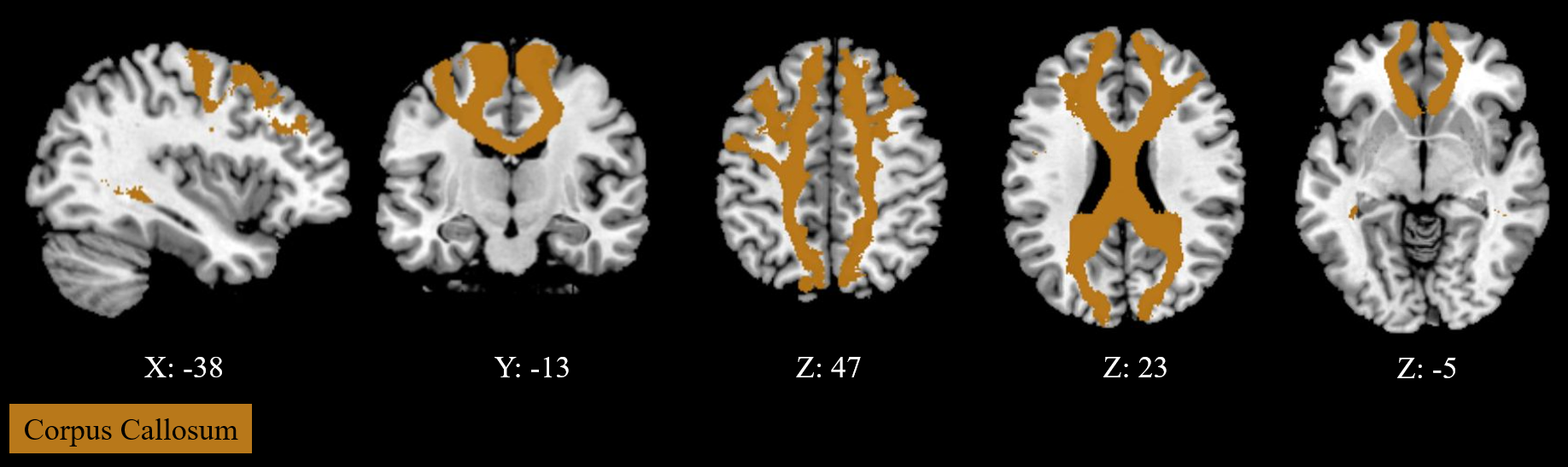 (k) – Corpus Callosum |

## *Supplementary Figure 5. (a) The mean probability of a given white matter tract being disconnected across the sample, and (b) the mean proportion disconnected. Generated using the Tractotron component of the BCB Toolkit (Foulon et al., 2018). Error bars reflect standard error of the mean. ATR = Anterior thalamic radiation, IFOF = Inferior fronto-occipital fasciculus, UF = Uncinate fasciculus, SLF = Superior longitudinal fasciculus, ILF = Inferior longitudinal fasciculus, AF = Arcuate fasciculus, FAT = Frontal aslant tract. The mean probability of disconnection is highest in the corpus callosum at .99, followed by the superior longitudinal fasciculus 2 at .96, the arcuate fasciculus long at .95, and the superior longitudinal fasciculus 3 at .94. The lowest probability of disconnection is in the uncinate fasciculus at .61. Mean estimated proportion disconnected peaks in the frontal inferior longitudinal fasciculus at .37, followed by the superior longitudinal fasciculus 3 at .32, and the anterior arcuate fasciculus at .30. The lowest estimated mean proportion disconnected is in the corpus callosum, at .05. (c) The structural disconnection overlap map for the sample, thresholded at 19 cases. Structural disconnection is left lateralised at this threshold and shows maximal overlap with the superior longitudinal fasciculus and inferior fronto-occipital fasciculus. Visualisations of the (d) ATR, (e) UF, (f) ILF, (g) FAT, (h) AF, (i) SLF, (j) IFOF, and (k) corpus callosum are presented. All tracts are confined to the left hemisphere. N = 23*

## *Supplementary Table 3. Main effects of network followed by Wilcoxon contrasts comparing the extent of both lesion and functional disconnection (percent of network impacted) between all functional networks of interest.*

| *Lesion* | | | | | | |
| --- | --- | --- | --- | --- | --- | --- |
| Network main effect | | **F(2.2, 47.7) = 10.1, *p* < .001, η_p_^2^ = .32*** | | | | |
|  | DMN | | Semantic | SCN | SCN & MDN | MDN |
| DMN |  | |  |  |  |  |
| Semantic | Z = -2.4, *p =* .186 | |  |  |  |  |
| SCN | **Z = -3.2, *p =* .015*** | | Z = -2.5, *p =* .137 |  |  |  |
| SCN & MDN | **Z = -3.5, *p =* .005*** | | Z = -1.9, *p* = .569 | Z = -1.3, *p* > 1 |  |  |
| MDN | **Z = -4.2, *p <* .001*** | | Z = -0.7, *p >* 1 | Z = -2.1, *p =* .333 | Z = -2.1, *p =* 358 |  |
| *Functional Disconnection* | | | | | | |
| Network main effect | | **F(1.7, 38.2) = 27.2, *p* < .001, η_p_^2^ = .55*** | | | | |
|  | DMN | | Semantic | SCN | SCN & MDN | MDN |
| DMN |  | |  |  |  |  |
| Semantic | **Z = -3.9, *p* < .001*** | |  |  |  |  |
| SCN | **Z = -3.9, *p* < .001*** | | **Z = -3.3, *p =* .008*** |  |  |  |
| SCN & MDN | **Z = -3.8, *p =* .002*** | | **Z = -3.1, *p =* .019*** | Z = -1.0, *p* > 1 |  |  |
| MDN | **Z = -3.0, *p* = .030*** | | Z = -1.8, *p =* .727 | Z = -2.5, *p =* .138 | **Z = -3.1, *p =* .019*** |  |

Note: Non-parametric contrasts reported due to violation of the normality assumption. Results in both sections Bonferroni corrected for ten comparisons. * = significant result. N = 23.

| 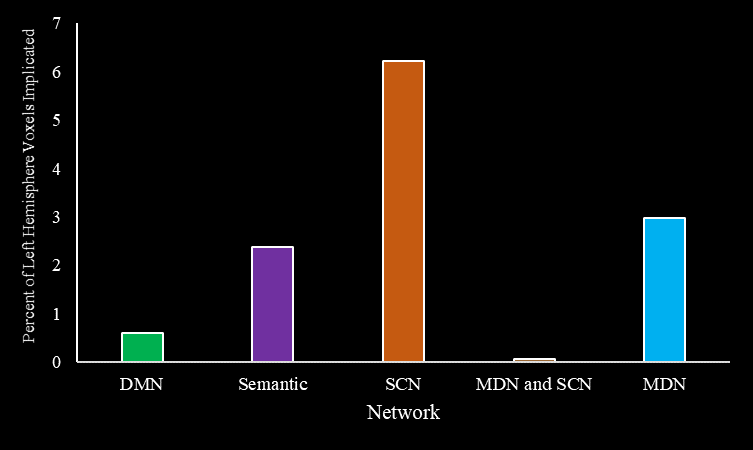 | 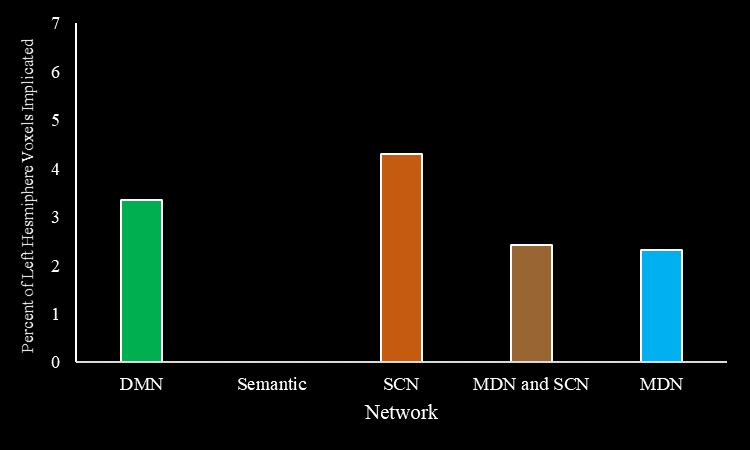 |
| --- | --- |
| 1. – Semantic Cognition Composite Score | 1. – Brixton Spatial Anticipation Test |

## *Supplementary Figure 6. The percentage of voxels in each network of interest, restricted to the left hemisphere, implicated in the group level lesion-symptom mapping output for (a) the semantic cognition composite score and (b) the Brixton Spatial Anticipation Test. DMN = default mode network, SCN = semantic control network, MDN = multiple demand network.^[[1]](#footnote-1)^ For the Semantic Cognition Composite Score, This peaks at 6.2% for the semantic control network, followed by 3.0% for the multiple demand network, 2.4% for core semantic regions, 0.6% for the default mode network, and 0.1% for areas shared by the semantic control and multiple demand networks. For the Brixton Spatial Anticipation Test, numbers peak in the semantic control network at 4.3%, followed by the default mode network at 3.6%, areas shared by the semantic control and multiple demand networks at 2.4%, and the multiple demand network at 2.3%, with 0% of core semantic regions implicated.*

# References

Bozeat, S., Lambon Ralph, M. A., Patterson, K., Garrard, P., & Hodges, J. R. (2000). Non-verbal semantic impairment in semantic dementia. *Neuropsychologia, 9*, 1207-1215. <https://doi.org/10.1016/s0028-3932(00)00034-8>.

Burgess, P. W., & Shallice, T. (1997). *The Hayling and Brixton Tests*. Bury St Edmunds: Thames Valley Test Company.

Corbett, F., Jefferies, E., & Lambon Ralph, M. A. (2011). Deregulated semantic cognition follows prefrontal and temporo-parietal damage: evidence from the impact of task constraint on nonverbal object use. *Journal of Cognitive Neuroscience, 23*(5), 1125-1135. <https://doi.org/10.1162/jocn.2010.21539>.

Fedorenko, E., Duncan, J., & Kanwisher, N. (2013). Broad domain generality in focal regions of frontal and parietal cortex. *PNAS, 110*(41), 16616-16621. <https://doi.org/10.1073/pnas.1315235110>.

Foulon, C., Cerliani, L., Kinkingnéhun, S., Levy, R., Rosso, C., Urbanski, M., Volle, E., & Thiebaut de Schotten, M. (2018). Advanced lesion symptom mapping analyses and implementation as BCBtoolkit. *GigaScience, 7*(3), 1-17. <https://doi.org/10.1093/gigascience/giy004>.

Jackson, R. L. (2021). The neural correlates of semantic control revisited. *NeuroImage, 224,* 117444. <https://doi.org/10.1016/j.neuroimage.2020.117444>.

Jefferies, E. (2013). The neural basis of semantic cognition: converging evidence from neuropsychology, neuroimaging and TMS. *Cortex, 49,* 611-625. <https://doi.org/10.1016/j.cortex.2012.10.008>.

Kay, J., Lesser, R., & Coltheart, M. (1992). *Psycholinguistic assessments of language processing in aphasia (PALPA)*. Hove (UK): Lawrence Erlbaum Associates.

Noonan, K. A, Jefferies, E., Corbett, F., & Lambon Ralph, M. A. (2010). Elucidating the nature of deregulated semantic cognition in semantic aphasia: Evidence for the roles of prefrontal and temporo-parietal cortices. *Journal of Cognitive Neuroscience, 22*(7), 1597-1613. <https://doi.org/10.1162/jocn.2009.21289>.

Raven, J. (1962). Coloured progressive matrices sets A, AB, B. London: H.K. Lewis.

Robertson, I., Ward, T., Ridgeway, V., & Nimmo-Smith, I. (1994). *The test of everyday attention*. London: Thames Valley Test Company.

Samson, D., Connolly, C., & Humphreys, G. W. (2007). When “happy” means “sad”: Neuropsychological evidence for the right prefrontal cortex contribution to executive semantic processing. *Neuropsychologia, 45,* 896-904. <https://doi.org/10.1016/j.neuropsychologia.2006.08.023>.

Thompson, H., Davey, J., Hoffman, P., Hallam, G., Kosinski, R., Howkins, S., Wooffindin, E., Gabbitas, R., & Jefferies, E. (2017). Semantic control deficits impair understanding of thematic relationships more than object identity. *Neuropsychologia, 104,* 113-125. <https://doi.org/10.1016/j.neuropsychologia.2017.08.013>.

Warrington, E. K., & James, M. (1991). *The Visual Object and Space Battery Perception*. Bury St Edmunds: Thames Valley Company.

Wechsler, D. (1997). *Wechsler memory scale (3rd ed.)*. San Antonio, TX: The Psychological Corporation.

Yeo, B. T. T., Krienen, F. M., Sepulcre, J., Sabuncu, M. R., Lashkari, D., Hollinshead, M., Roffman, J. L., Smoller, J. W., Zöllei, L., Polimeni, J. R., Fischl, B., Liu, H., Buckner, R. L. (2011). The organization of the human cerebral cortex estimated by intrinsic functional connectivity. *Journal of Neurophysiology, 106*(3), 1125-1165. <https://doi.org/10.1152/jn.00338.2011>.

1. Note that these percentages will be impacted by differences in the relative size of each network. The number of voxels implicated over the total size of the respective number of voxels in each network for the Semantic Cognition Composite Score is: DMN: 82/13,618, Semantic: 84/3,549, SCN: 220/3,538, MDN & SCN: 1/1,777, MDN: 380/12,731. For the Brixton Spatial Anticipation Test, it’s: DMN: 457/13,618, Semantic: 0/3,549, SCN: 152/3,538, MDN & SCN: 43/1,777, MDN: 296/12,731. Due to these differences in size, comparisons for a give network between graphs will be most informative. [↑](#footnote-ref-1)
