# Supplementary material for: Mapping lesion, structural disconnection, and functional disconnection to symptoms in semantic aphasia
Source: Brain Struct Funct. 2022 Jul 4;227(9):3043–61. doi: 10.1007/s00429-022-02526-6 (PMC9653334; doi:10.1007/s00429-022-02526-6)
Supplement: Supplementary file 2 — Supplementary file2 (DOCX 12 KB) [file 429_2022_2526_MOESM2_ESM.docx]

Mapping lesion, structural disconnection, and functional disconnection to symptoms in semantic aphasia – Links to Supporting Data

Open Science Framework:

<https://osf.io/6psqj/>

Neurovault:

<https://neurovault.org/collections/KGXBJXSX/>
